# Structural and Morphological Characterization of Gd-Doped Ceria (Ce_1−x_Gd_x_O_2−x/2_) Synthesized by an Optimized Hydrothermal Method

**DOI:** 10.3390/ma18214957

**Published:** 2025-10-30

**Authors:** Kolyo Kolev, Ognian Dimitrov, Mariela Dimitrova, Maria Shipochka, Daniela Karashanova, Tamara Petkova

**Affiliations:** 1 Institute of Electrochemistry and Energy Systems, Bulgarian Academy of Sciences, Acad. G. Bonchev Str., bld.10, 1113 Sofia, Bulgaria; ognian.dimitrov@iees.bas.bg (O.D.); mariela.dimitrova@iees.bas.bg (M.D.); tpetkova@iees.bas.bg (T.P.); 2 “National Centre of Excellence Mechatronics and Clean Technologies”, 8 bul. Kliment Ohridski, 1756 Sofia, Bulgaria; 3 Institute of General and Inorganic Chemistry, Bulgarian Academy of Sciences, Acad. G. Bonchev Str., bld.11, 1113 Sofia, Bulgaria; shipochka@svr.igic.bas.bg; 4 Institute of Optical Materials and Technologies “Acad. Jordan Malinowski”, Acad. G. Bonchev Str., bld.109, 1113 Sofia, Bulgaria; dkarashanova@yahoo.com

**Keywords:** CeO_2_, ceria, Gd-doped CeO_2_, rare-earths, hydrothermal synthesis, nanoparticles

## Abstract

The aim of the presented work is to develop a more energy- and time-saving modification of a well-known hydrothermal synthesis method by reducing the time of the synthesis regime and drying step, as well as the possible removal of the calcination procedure. The structure and morphology of Gd-doped ceria (Ce_1−x_Gd_x_O_2−x/2_, where x = 0, 0.1, 0.2, 0.3, and 0.5), synthesized via the optimized hydrothermal method, were thoroughly investigated. Phase composition was analyzed using X-ray diffraction (XRD), while the structural units of the materials were identified by Fourier-transform infrared spectroscopy (FTIR). Chemical composition was studied using energy-dispersive X-ray spectroscopy (EDS) and further confirmed by energy-dispersive X-ray fluorescence (EDXRF). Transmission electron microscopy (TEM) was employed to analyze the size and shape of the nanoparticles. Raman spectroscopy and X-ray photoelectron spectroscopy (XPS) confirmed the presence of Ce^3+^ ions in both doped and undoped CeO_2_ samples.

## 1. Introduction

Increasing demand for sustainable technologies and environmental protection has led to the development of multifunctional oxide materials with tunable structural and surface properties. Cerium oxide (CeO_2_) and its doped derivatives have attracted considerable attention due to their outstanding redox flexibility, oxygen storage capacity, and defect-driven ionic and electronic transport. These characteristics make ceria-based materials suitable for diverse applications, including heterogeneous and photocatalysis, wastewater treatment, gas sensing, corrosion protection, and solid-state electrochemical devices, as well as biomedical uses such as antioxidant and antimicrobial systems [1,2,3,4,5,6,7,8,9,10,11].

The exceptional functionality of ceria arises from its ability to easily form and migrate oxygen vacancies. However, the efficacy of pure CeO_2_ in redox and electrochemical processes is limited by its comparatively poor ionic conductivity [12]. A proven strategy to overcome this limitation involves doping with trivalent cations (M^3+^), which substitute Ce^4+^ and create charge-compensating oxygen vacancies that enhance ionic and electronic transport. Among the various dopants, gadolinium (Gd^3+^) has shown the most effective improvement in conductivity and stability, owing to its ionic radius (1.053 Å) being close to that of Ce^4+^ (0.97 Å) [13,14,15]. As a result, Gd-doped ceria (Ce_1−x_Gd_x_O_2−x/2_, GDC) has become a model system for studying defect chemistry and structure–property relationships in multifunctional oxides.

The performance of GDC strongly depends on the synthesis method, which influences crystallinity, particle size, morphology, and defect concentration. A number of works are dedicated to the synthesis of Gd-doped ceria via various techniques like the Pechini method [16] and the polymeric precursor [17], co-precipitation [15], combustion [18], solid-state [12], ionic gelation [19], and hydrothermal [20] methods. Among them, hydrothermal synthesis remains one of the most efficient and environmentally friendly approaches. It facilitates direct crystallization from aqueous solution under moderate conditions, permitting fine control over particle characteristics by modifying precursor composition, pH, temperature, and reaction time. The absence of organic solvents and the use of water-soluble reagents further enhance its sustainability.

In this study, an optimized hydrothermal synthesis route for Gd-doped ceria was developed with the aim of obtaining materials with high crystallinity and predictable morphology under significantly reduced synthesis and drying times. Compared to conventional processes involving approximately 24 h of synthesis followed by 12–24 h of drying [4,20,21,22,23], the proposed optimized route reduces the synthesis and drying stages to 5 and 10 h, respectively, and explores the elimination of the calcination step, assuming that the increased drying temperature can fulfill its role. The primary motivation for this optimization is to accomplish the entire relevant process (synthesis, drying) within a single working day, thereby reducing energy consumption and yielding potential economic and environmental benefits while maintaining the desired material quality.

Extensive structural and morphological characterization by XRD, FTIR, TEM, EDS, EDXRF, Raman, and XPS was performed to assess the effects of synthesis modification on crystallinity, defect formation, and surface composition. The results provide insight into the controlled preparation of Gd-doped cerium oxide and demonstrate its potential as a sustainable multifunctional oxide for catalytic, environmental, and electrochemical applications.

## 2. Materials and Methods

Gd-doped ceria with the composition of (Ce)_1−x_(Gd)_x_(O)_2−x/2_ (x = 0–0.5) was obtained from cerium nitrate (Ce(NO_3_)_2_·6H_2_O, Acros Organics, Geel, Belgium, 99.9%) and the corresponding amount of Gd(NO_3_)_3_·6H_2_O (Acros Organics, 99.9%). In total, 0.02 mol of the nitrates in the corresponding proportions were each dissolved in 20 mL of distilled water, followed by the rapid addition of 30 mL 5 M NaOH solution (99%, Chem-lab NV, Zedelgem, Belgium) under vigorous stirring. The mixture was agitated for about 5 min and then placed in a 100 mL Teflon vessel reaching 80% of its volume, which was sealed and transferred to the hydrothermal system Berghof BR-100, Eningen, Germany. The solution was subjected to hydrothermal synthesis at 180 °C for 5 h at a heating rate of 3 °C.min^−1^, followed by air cooling to room temperature. The obtained precipitate was separated from the solution by vacuum filtration and washed several times with distilled water and ethanol to reach pH = 7, then dried at 120 °C for 8 h. Finally, the samples were separated into two groups, one of which was calcinated at 500 °C for 3 h. The composition/abbreviation of the synthesized samples is presented in Table 1.

The phase composition of the samples was studied with the X-ray diffractometer PANalytical Aeris with Cu-Kα radiation (λ = 1.5406 Å) and θ-θ Bragg–Brentano geometry. The diffractograms were obtained at room temperature, constant scan rate, and reflection angle 2θ in the interval 5–80° with a step of 0.02° and interpreted with the help of the PDF 2-2022 database. The lattice parameters were determined with the Rietveld method by fitting the shape and position of the peaks from the X-ray diffraction patterns. The average crystallite size (D) of the obtained materials was calculated using the Debye–Scherrer equation:

(1) D=0.9λ/βcosθ

where λ is the wavelength of the X-rays (1.5418 Å), θ is the scattering angle of the main reflection (111), and β is the corrected peak at full width at half-maximum (FWHM) intensity.

Raman spectra were collected on a Renishaw InVia Qontor Raman with Confocal Microscope, Gloucestershire, UK, equipped with a 532 nm laser. Data were collected from powder samples at room temperature in the frequency range 3000–100 cm^−1^, with laser power kept at ~2 mW, processed via the software WiRE 5.5.

X-ray photoelectron spectroscopy (XPS) studies were performed by a VG ESCALAB II electron spectrometer, Waltham, MA, USA, using AlKα radiation with an energy of 1486.6 eV. The binding energies (BEs) were determined with an accuracy of ±0.1 eV utilizing the C1s line at 285.0 eV from an adventitious carbon as a reference. The changes in composition and chemical surrounding in the depth of the powders were determined on the basis of the areas and binding energies of C1s, O1s, Ce3d, and Gd4d photoelectron peaks (after linear subtraction of the background) and Scofield’s photoionization cross-sections.

Fourier-transform infrared (FTIR) spectroscopy was applied to determine the molecular structure of the samples using an Invenio-Bruker FTIR spectrophotometer, Rosenheim, Germany. All measurements were performed at room temperature in the 4000–200 cm^−1^ range at 2 cm^−1^ resolution. Each spectrum was recorded after 100 scans.

The chemical composition and elemental mapping of the samples was studied with a scanning electron microscope (SEM) Zeiss Evo 10 (Carl Zeiss Microscopy, Jena, Germany), equipped with the electron-dispersive X-ray spectroscopy (EDS) probe Oxford Ultim Max 40 (Oxford Instruments, Abingdon, UK). The results were processed with Aztec software (version 6.1 HF4).

For elemental analysis, Epsilon 1 energy-dispersive X-ray fluorescence (EDXRF) analyzer (PANalytical, Malvern, UK) with an Ag X-ray tube at 50 kV voltages and 10 W maximum power was applied. The powders were studied by transmission electron microscopy (TEM) using a high-resolution transmission electron microscope JEOL JEM 2100 (JEOL Ltd., Tokyo, Japan) at 200 kV accelerating voltage. The microstructure and the phase composition of the particles were studied by the diffraction modes of the microscope—selected area electron diffraction (SAED) and high-resolution TEM (HRTEM).

Under the applied hydrothermal conditions (180 °C, 5 h), the conversion of the cerium nitrate precursor to cerium oxide was complete as confirmed by the absence of residual cerium hydroxide or nitrate phases in the XRD and FTIR spectra. XPS and EDXRF analyses additionally confirmed the expected Ce/Gd stoichiometry and the coexistence of Ce^4+^/Ce^3+^ oxidation states typical of CeO_2−x_, indicating nearly 100% precursor conversion.

## 3. Results and Discussion

**Figure 1:**
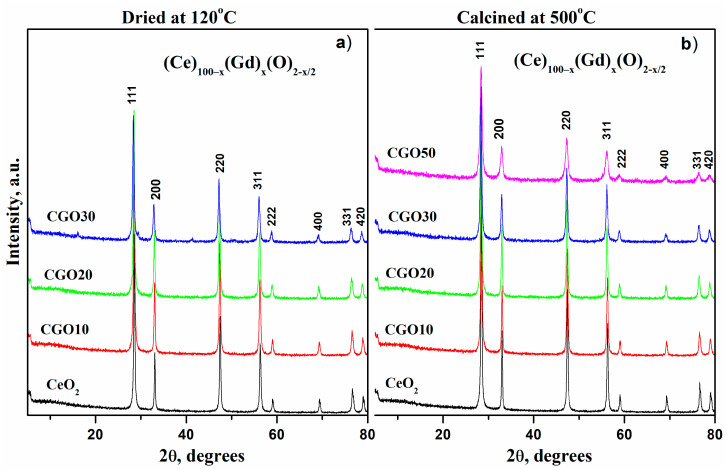
XRD patterns of (Ce)_1−x_(Gd)_x_(O)_2−x/2_ systems, dried at 120 °C (**a**) and calcined at 500 °C (**b**).

The XRD patterns of the Ce_1−x_Gd_x_O_2−x/2_ (0 ≤ x ≤ 0.5) dried and calcinated samples are shown in Figure 1a and 1b, respectively. The major characteristic planes of Gd-doped ceria are observed at (111), (200), (220), (311), (222), (400), (331), and (420) [14,22,23]. Using the PDF-2 2022 database for diffractogram interpretation, the ICDD reference pattern for Cerianite (ICSD 98-002-9046; ICDD PDF #01-073-6328), which crystallizes in the fluorite-type (CaF_2_) structure, was employed as the initial structural model.

Upon substitution with gadolinium in the ceria lattice up to x = 0.3, the XRD patterns of the studied samples clearly prove the presence of a cubic fluorite structure (Fm-3m) showing no changes in the lattice symmetry. The sample containing 50% Gd has a cubic Ia-3 structure (JCPDS card no. 80-6918).

No peaks of pure Gd_2_O_3_, CeO_2_ or Ce_2_O_3_ phases are observed in the diffraction pattern, only that of the mixed phase (Ce)_1−x_ (Gd)_x_(O)_2−x/2_, confirming solubility of the gadolinium in the cerium lattice. The most intense peak (111) decreases significantly and becomes broader as the gadolinium content rises, likely due to the substitution of Ce with a heavier atom. This suggests an increased absorption factor, leading to reduced intensity in transmission mode.

**Figure 2:**
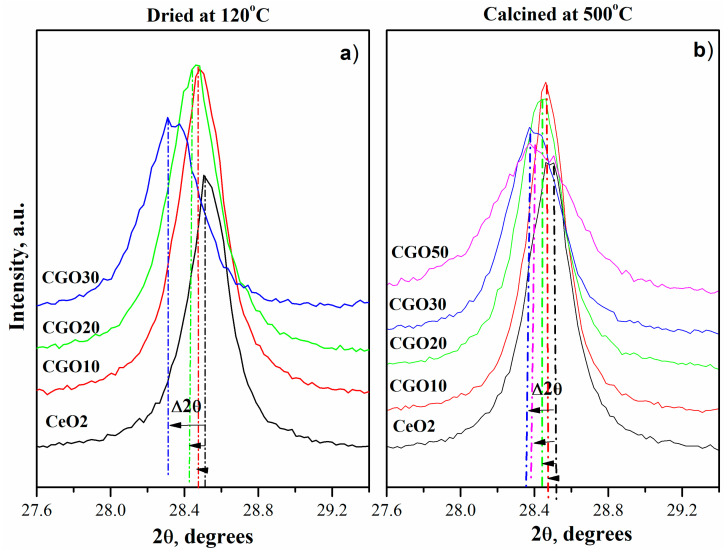
XRD patterns of (Ce)_1−x_(Gd)_x_(O)_2−x/2_ systems in the 2θ range around the (111) reflection, dried at 120 °C (**a**) and calcined at 500 °C (**b**).

Although the diffractograms of pure CeO_2_ and Gd-doped CeO_2_ (10–50%) appear to completely overlap at first glance, a closer inspection reveals a slight shift of the diffraction peaks toward smaller angles, associated with an increase in interplanar spacing. For clarity, Figure 2 presents the diffraction patterns in a narrower 2θ range around the (111) reflection, before and after gadolinium incorporation. The peak shifts to lower 2θ values (from 28.55° to 28.29°), corresponding to an increase in the interplanar spacing from 3.12 to 3.15 Å. This trend is disrupted at the 50:50 Ce/Gd ratio, which exhibits less shift than the 70:30 Ce/Gd ratio. These results are consistent with the data reported in the PDF-2 crystallographic database: Ce_0.90_Gd_0.10_O_1.95_ (#01-086-9063), Ce_0.80_Gd_0.20_O_1.90_ (#01-080-5535), Ce_0.70_Gd_0.30_O_1.85_ (#01-084-3514), and Ce_0.50_Gd_0.50_O_1.75_ (#01-084-3518).

The narrow, intense diffraction peaks indicate good crystallinity of the materials after drying. However, the elevated background at low angles suggests the presence of an amorphous phase. A comparative analysis of peak shape was performed to further assess this. The analysis involved calculating the full width at half-maximum (FWHM) and the width at the top (“tip width”), a parameter derived from HighScore Plus software (version 5.2 (5.2.0.31529)). The relative difference (ratio%) between the two values, calculated as (tip width − FWHM)/FWHM × 100, indicates sharp peaks which are consistent with a high degree of crystallinity (Table 2). Although “tip width” is not a standard diffraction parameter, it provides qualitative information about peak symmetry and sharpness. This information complements both the visual and quantitative evaluation of crystallinity.

The degree of crystallinity (%χ) was also calculated by integrating the area under the crystalline peaks relative to the total area. The results presented in Table 2 range from 37% to 46%. This is lower than expected from the peak-width analysis, which indicates the possible presence of microstructural defects or stresses. The relatively low degree of crystallinity does not necessarily imply a high amorphous content. In solid solutions, especially those containing cations of different ionic radii, structural distortions and microstrain can produce diffuse scattering and background broadening. These effects contribute to the total diffracted area and may be interpreted as an “amorphous” fraction, even though the material remains predominantly crystalline at the nanoscale. The presence of sharp diffraction peaks and their systematic shift with composition confirm the formation of a well-developed solid solution with localized lattice distortions rather than a truly amorphous phase.

The lattice parameters and crystallite size of the samples presented in Table 3 were obtained using the Rietveld refinement method. The data clearly show an increase of the cell parameter with increasing Gd content attributed to the substitution of smaller Ce^4+^ ions with larger Gd^3+^ ions [22]. At the same time, the volume of the unit cell changes slightly after calcination at 500 °C (0.05–0.24 Å^3^). A decrease in the crystalline size is also observed as the gadolinium amount rises.

A noticeable change in indexed symmetry occurs from a body-centered cubic model at 120 °C to a face-centered (fluorite) model at 500 °C. This change may arise from incomplete crystallization, a defective fluorite structure, or the formation of oxygen vacancies. When the Ia-3 parameters are normalized to the same fluorite-type unit cell, the lattice parameters of the dried and calcined samples differ only slightly (Δa ≈ 0.0002 Å), confirming that the structural transformation is associated with cation ordering and lattice relaxation rather than a doubling of the cell size. While these explanations support the effect of calcination, the Rietveld-refined microstrain values (Table 4) provide a quantitative measure of lattice disorder and further insight into the structural evolution of the CeO_2_–Gd_2_O_3_ system. The values increase markedly with Gd content, from 9.41 × 10^−6^ (pure CeO_2_) to 1.83 × 10^−3^ (CGO50) after calcination.

**Figure 3:**
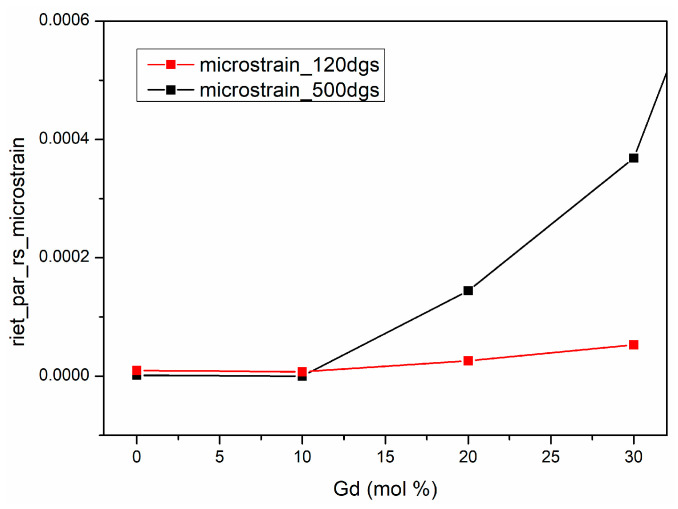
Microstrain (ε) variation of Ce_1−x_Gd_x_O_2−x/2_ samples as a function of Gd content, before and after calcination.

The low microstrain values for CeO_2_ and CGO10 indicate structural relaxation and removal of residual hydroxides upon heating, whereas the pronounced rise for CGO_20_–CGO_50_ reflects substantial lattice distortion due to Gd^3+^ substitution and oxygen-vacancy formation (Figure 3). These microstrain-induced effects explain the observed Ia-3 space group for the dried CGO30 sample and the increased XRD background, confirming earlier conclusions regarding the relatively low apparent crystallinity obtained from integrated peak areas. The extremely high microstrain (~10^−3^) in CGO50 indicates a highly distorted crystal lattice and describes the transition toward a bixbyite-type structure ((Mn,Fe)_2_O_3_-type), involving the formation of Gd_2_O_3_-like domains. This is consistent with the observed changes in phase symmetry and confirms that calcination does not improve crystallinity but rather increases internal stresses.

Importantly, samples dried at 120 °C and those dried then calcined at 500 °C exhibited similar crystallite sizes, unit cell parameters, and crystallinity values (a difference of only 0.45–1.23%), demonstrating that calcination does not meaningfully improve crystallinity substantially and is therefore economically unjustified.

**Figure 4:**
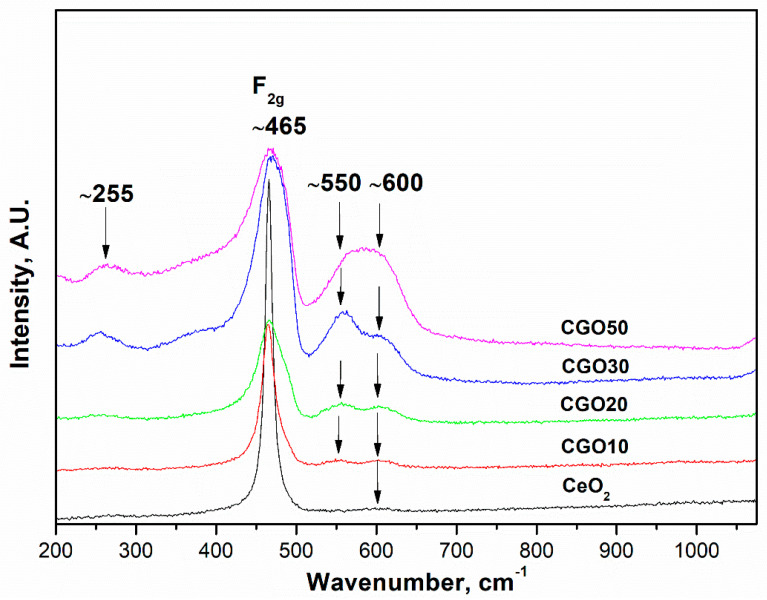
Raman spectra of (Ce)_1−x_(Gd)_x_(O)_2−x/2_ system calcined at 500 °C. The y axis is shifted in all spectra for better viewing purposes.

The Raman spectra of pure and Gd-doped samples are shown in Figure 4. The spectrum of pure CeO_2_ consists of one main band at ~465 cm^−1^ and a very slight hump at ~600 cm^−1^. With the introduction of Gd^3+^ ions, a new Raman band at ~540 cm^−1^ appears for the compositions with 10 and 20% Gd content. Along with the new band, the main Raman band at ~465 cm^−1^ widens and reduces in intensity with pronounced asymmetry. According to Acharya et al. [24], the reason for this is the lattice expansion induced by the substitution of Ce^4+^ ions with Gd^3+^ ions. Further increase of Gd content to 30 and 50% leads to the emerging of a new Raman band at ~255 cm^−1^.

The band at ~465 cm^−1^ is ascribed to the F_2g_ symmetric breathing mode of the Ce−O bond in eightfold coordination [25]. The bands at 600, 550, and 255 cm^−1^ are referred to as disorder bands (or D bands) and are defect-induced Raman signals [17,24,26,27,28]. The broad band around 600 cm^−1^ can be attributed either to the introduction of a doping cation forming a REO_8_-type complex that does not contain any oxygen vacancies or to the presence of intrinsic oxygen vacancies induced by a certain amount of Ce^3+^ [17,24,26]. The presence of the slight hump at ~600 cm^−1^ even in the pure CeO_2_ allows us to assume that there are defects resulting from existing Ce^3+^ in the lattice of undoped CeO_2_ [24].

The introduction and increased concentration of Gd^3+^ ions result in the emergence of additional D bands at approximately 255 and 550 cm^−1^. These bands are attributed to the formation of oxygen vacancies and their interactions with the six nearest neighboring oxygen atoms [26].

According to a number of authors, defect sites are morphology dependent [29,30]. It is known that the vacancy formation energy of nanorods is lower than that of nanocubes, so we should expect more defect states in nanorods than in nanocubes [31]. And our results presented in Figure 2 correspond to this observation since we observe increasing D bands with the morphology shift from nanocubes to nanorods (as shown in the HRTEM pictures presented below).

Also, we have to note that there is no evidence for a vibrational mode corresponding to the Gd_2_O_3_ phase, expected at 375 cm^−1^ [32]. This observation confirms the formation of Gd-doped ceria solid solutions in corroboration with our XRD data.

**Figure 5:**
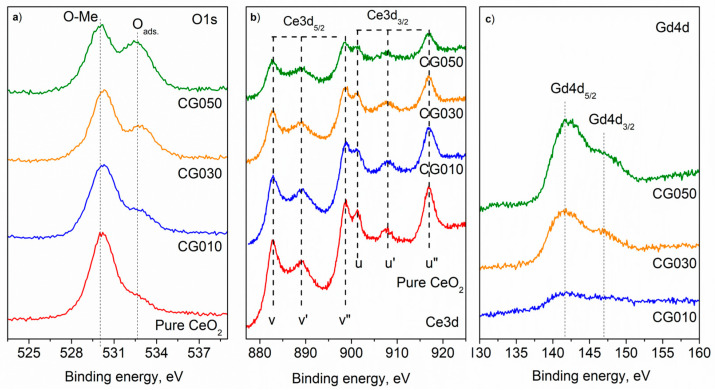
Photoelectron spectra of (**a**) O1s, (**b**) Ce3d, and (**c**) Gd4d of the powders.

The XPS results presented in Figure 5 show the presence of the chemical elements O, Ce, and Gd on the surface (Table 5).

Two well-resolved peaks are observed in the oxygen spectra. The more intense peak at higher binding energy corresponds to oxygen bound to metals, while the less intense peak is attributed to oxygen adsorbed on the surface. The spectra of Ce 3d consist of six peaks—v, v′, and v″ and u, u′, and u″—for doublet separation of Ce 3d_5/2_ and Ce 3d_3/2_ (Figure 5b). The shape of the Ce 3d spectra, and in particular the u″ peak at binding energy 917 eV, is attributed to the Ce^4+^ oxidation state [33]. The binding energy at 141.7 eV for Gd 4d_5/2_ and 147 eV for Gd 4d_3/2_, as well as the spin-orbit splitting from 5.3 eV between them, refers to Gd–O interactions [34].

**Figure 6:**
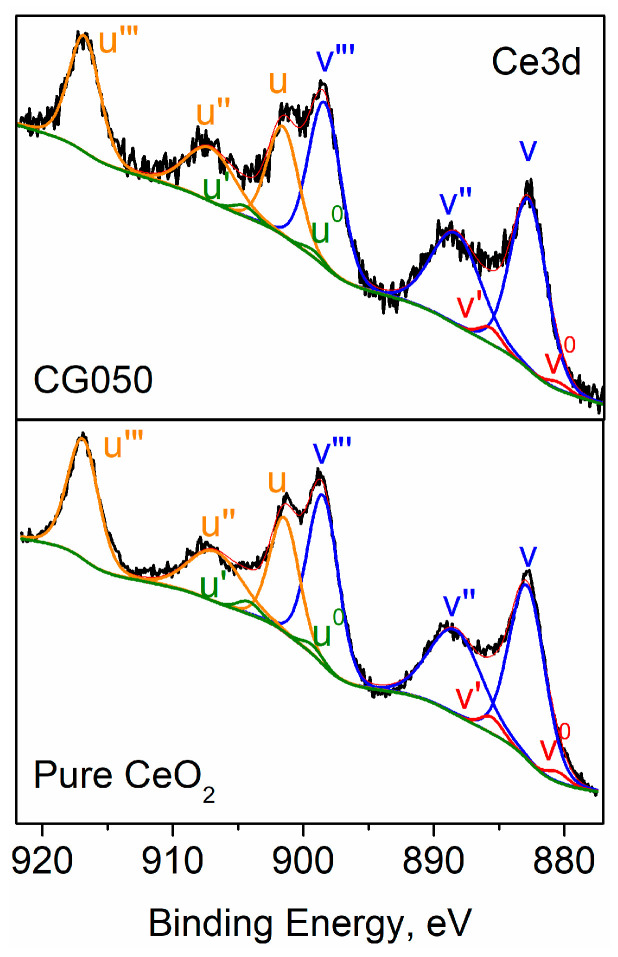
Decomposed photoelectron spectra for pure CeO_2_ and CGO50.

However, after calculating the ratio between the areas of the u″ peak and the area of the entire Ce 3d region, it is found that a small amount of Ce^3+^ is also present. The amounts of Ce^3+^ and Ce^4+^ were calculated using the following equation:

(2) $${Ce}^{4+}\%=\left(U^{\prime \prime \prime }\%/14\right)\times 100\%$$

where U‴% represents the relative area of the U‴ peak with respect to the total Ce3d spectral area. The pure cerium and the cerium sample containing 50% Gd exhibit the highest amounts of Ce^3+^ among all the samples, with 2.7% and 2.0%, respectively [35]. From the areas of the decomposed peaks assigned to Ce^3+^ and Ce^4+^, the Ce^3+^/Ce^4+^ ratio was calculated to be 0.06 for pure cerium and 0.04 for the 50% Gd sample. The decomposed spectra are presented in Figure 6.

These results indicate that the material is almost completely oxidized to CeO_2_, with only minimal reduction to Ce. The observed presence of the Ce^3+^ oxidation state is in good agreement with the results of the Raman investigation.

**Figure 7:**
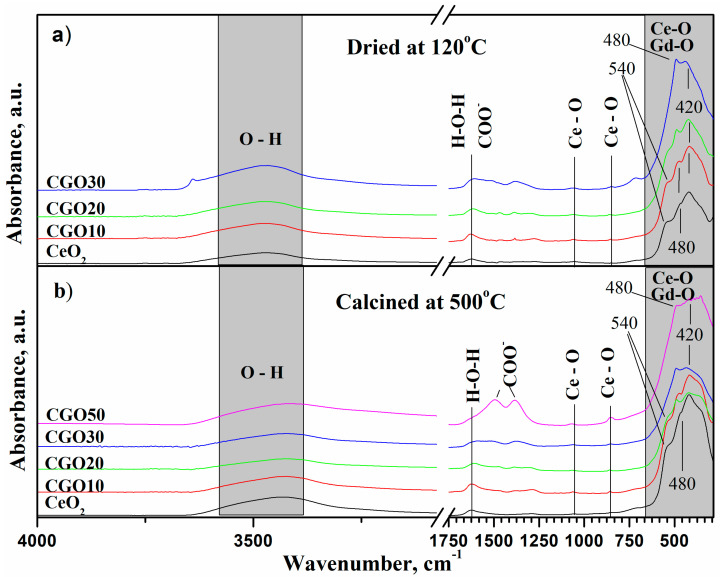
Infrared spectra of the (Ce)_1−x_(Gd)_x_(O)_2−x/2_ system of materials (**a**) dried at 120 °C and (**b**) calcined at 500 °C.

The infrared spectra of the dried and calcined Gd-doped ceria powders are shown on Figure 7. The broad band around ~3400 cm^−1^ is characteristic of the O–H stretching vibrations of water molecules. The band at ~1630 cm^−1^ corresponds to the deformation vibrations of H–O–H in water molecules, but it may also overlap with C=O stretching vibrations [12,22,36]. The peaks at 1060 cm^−1^ and 850 cm^−1^ belong to the Ce–O group and Ce–O–Ce asymmetric valence vibrations, respectively. Peaks below 550 cm^−1^ are characteristic of Me–O (M = Ce, Gd) valence vibrations.

The reduction of the shoulder at ~540 cm^−1^, the transformation of the feature at ~480 cm^−1^ from a shoulder into a distinct peak, and the corresponding decrease in the intensity of the ~420 cm^−1^ peak could be related to the increased Gd content and the formation of Ce–O–Gd bonds [37,38].

**Figure 8:**
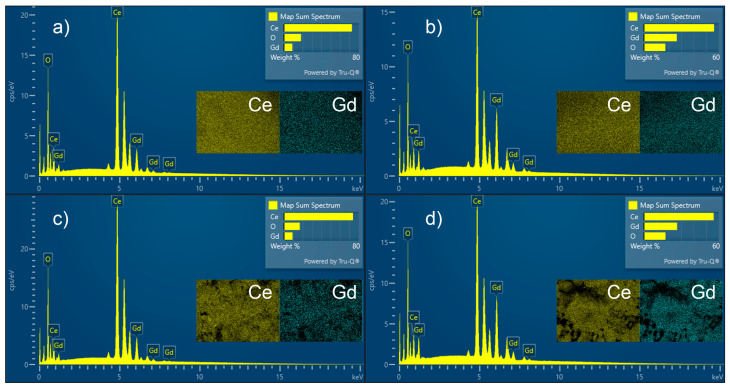
EDS spectra with elemental maps insets of samples CGO10 (**a**) and CGO30 (**b**) dried at 120 °C and samples with the same composition CGO10 (**c**) and CGO30 (**d**) calcined at 500 °C.

Electron-dispersive X-ray spectroscopy (EDS) was deployed to study the elemental composition of the powders. The selected spectra presented in Figure 8 compare samples with the same composition (10% and 30% Gd), synthesized under different temperature regimes (120 °C and 500 °C). The results reveal no change in the shape or position of the peaks, only a slight increase of their intensity for the annealed samples. The elemental mapping insets show that Ce and Gd are uniformly and homogeneously distributed throughout the materials. This confirms the efficiency of the selected synthesis procedure.

Table 6 presents the elemental composition of the samples examined by electron-dispersive X-ray spectroscopy (EDS) and energy-dispersive X-ray fluorescence (EDXRF). The calculated percentages for Ce and Gd are in good agreement with the assigned compositions.

**Figure 9:**
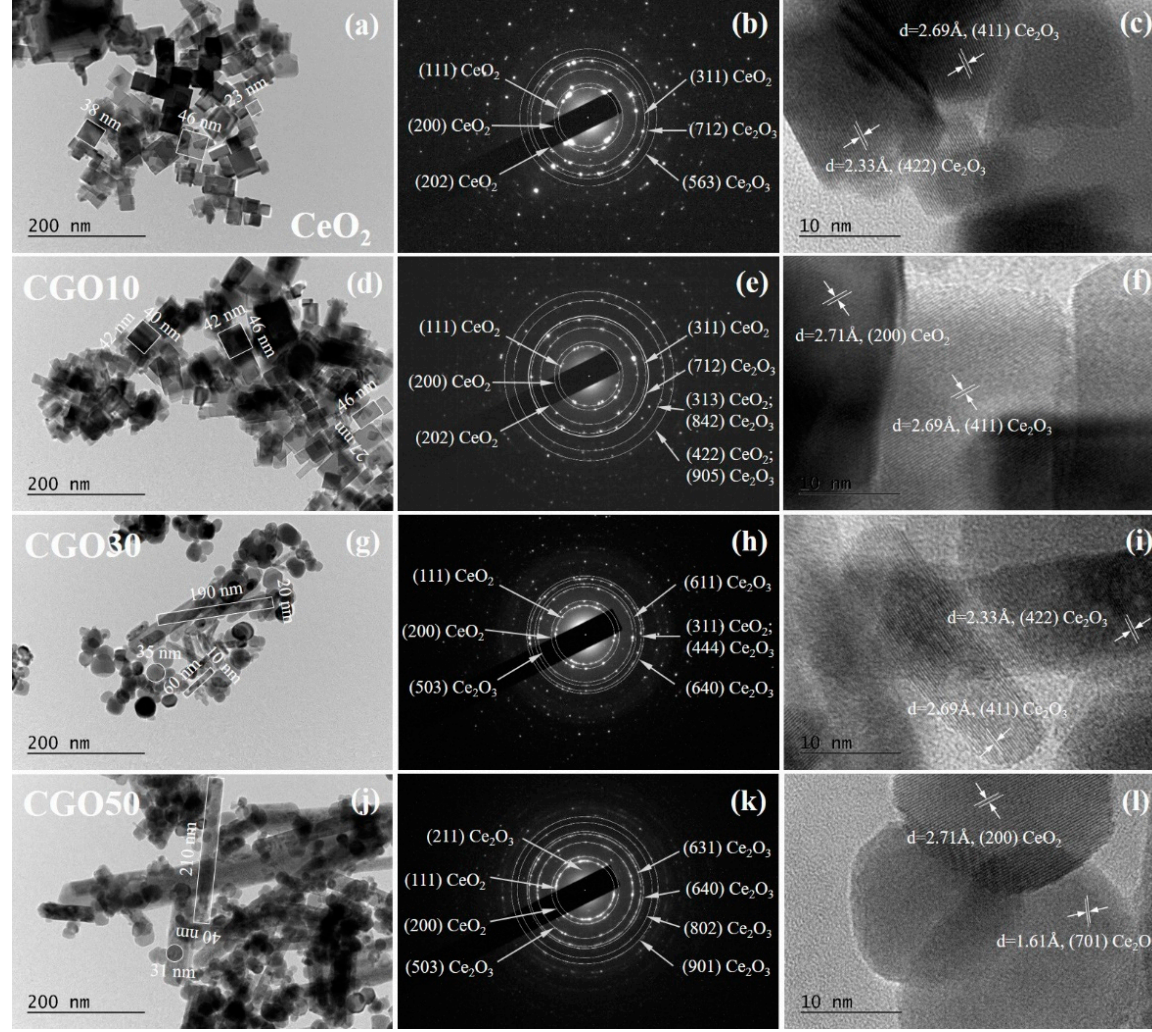
Bright-field TEM micrographs (**a**,**d**,**g**,**j**), electron diffraction (SAED) patterns (**b**,**e**,**h**,**k**), and HRTEM (**c**,**f**,**i**,**l**) of the calcined samples: (**a**–**c**) CeO_2_; (**d**–**f**) CGO10; (**g**–**i**) CGO30; (**j**–**l**) CGO50.

The BF TEM micrographs shown on Figure 9 reveal well-dispersed non-agglomerated nanoparticles with defined morphology [39]. A clearly visible transformation in the shape and size of the nanosized particles and nanorods’ growth is established with increasing Gd content. It is observed that pure CeO_2_ forms cuboidal particles, whose cross-sections in the image plane are almost perfect squares with different side lengths of the order of 20–40 nm (Figure 9a). Very rarely they are rectangular-shaped, but rather some of them have stuck together during drying on the TEM grid, touching their walls. This morphology of CeO_2_ is different from the reported in [39] with nanorods of pure CeO_2_, which could be due to the difference in the experimental parameters and preparation conditions used in this study—namely (i) the use of nitrates as precursors, (ii) the high NaOH concentration (pH above 9), (iii) the high synthesis temperature of 180 °C, and (iv) a sufficient reaction time [40,41].

When 10 mol. % Gd, a representative rare earth element, is included in the composition (sample CGO10), a deviation from the square shape can be detected in many of the particles, as well as the presence of rods that begin to form in the final oxide (Figure 9d), stimulated by the anisotropy in the growth of CeGd(OH)_3_ during the synthesis of the compound, as discussed in [17,39]. As an intermediate phase, Gd(OH)_3_ exhibits a tendency to grow along the one-dimensional (001) direction, and this intrinsic property of Gd to form 1D nanostructures promotes the anisotropic growth of cerium nuclei. The drying process does not reverse this preferential growth, resulting in the formation of nanorods [39]. In the compositions with 30 and 50 mol % Gd (samples CGO30 and CGO50), the nanorods are well expressed with sizes of the order of 50–200 nm and exceeding 200 nm, respectively. In these two compositions, along with the nanorods, the presence of spherical-like or irregular particles is noticeable, the size of which is of the order of 20–40 nm for CGO30 (Figure 9g), becoming even smaller for CGO50 (Figure 9j). The appearance of this second morphology, together with the nanorods, could be due to the fact that these compositions are close to and above the upper solubility limit of Gd in CeO_2_ (x = 0.30) and CeO_2_ nanorods are not the only morphology in the solid solutions [17].

In all samples, substitution of Ce^4+^ ions with trivalent Gd^3+^ leads to the formation of Ce^3+^ ions and oxygen vacancies [7,19], which may account for the presence of a Ce_2_O_3_ phase observed in HRTEM images and SAED patterns. Although the amount of this phase is too small to be detected by XRD, its presence is supported by Raman and XPS analyses which confirm the existence of Ce^3+^.

## 4. Conclusions

Gd-doped ceria with composition (Ce)_1−x_(Gd)_x_(O)_2−x/2_ (where x = 0, 0.1, 0.2, 0.3, and 0.5) was synthesized via an optimized, simplified hydrothermal method. XRD and FTIR analyses reveal that at up to 50% Gd incorporation, no significant structural changes occur as evidenced by the absence of additional diffraction peaks in the XRD patterns and new bands in the IR spectra. The fluorite-type lattice remains stable up to 50% Gd content, where a structural transformation is observed, indicated by a change in the space group from Fm-3m to Ia-3. Samples with and without thermal treatment at 500 °C exhibited similar crystallite sizes, unit cell parameters, and crystallinity values, demonstrating that calcination does not meaningfully improve crystallinity. Raman spectra show a single dominant F_2_g band characteristic of CeO_2_, with no significant shift observed as Gd content increases. The oxygen vacancies identified are due to intrinsic vacancies resulting from the partial reduction of Ce^4+^ to Ce^3+^ and extrinsic vacancies introduced by trivalent Gd doping. Elemental analysis by both EDS and EDXRF verifies that the compositions align with the intended stoichiometry. TEM analysis reveals a morphological transition from CeO_2_ nanocubes to irregular nanoparticles and nanorods upon Gd doping. XPS data further confirm the presence of Ce^3+^. In summary, Gd-doped ceria nanopowders were successfully synthesized via an optimized hydrothermal route using environmentally benign precursors and simplified processing steps. The approach allowed precise control of phase purity, crystallinity, and particle morphology while significantly reducing synthesis time and energy consumption. Comprehensive structural, morphological, and compositional analyses confirmed the complete conversion of precursors and the formation of single-phase fluorite-type Ce_1−x_Gd_x_O_2−x_/_2_ solid solutions.

## Figures and Tables

**Table 1:** Composition/abbreviation of synthesized samples used in text.

Composition of (Ce)_1−x_(Gd)_x_(O)_2−x/2_	Abbreviation
CeO_2_	CeO_2_
Ce_0.9_Gd_0.1_O_1.95_	CGO10
Ce_0.8_Gd_0.2_O_1.90_	CGO20
Ce_0.7_Gd_0.3_O_1.85_	CGO30
Ce_0.5_Gd_0.5_O_1.75_	CGO50

**Table 2:** Degree of crystallinity of (Ce)_1−x_(Gd)_x_(O)_2−x/2_ system samples.

Sample	120 °C				500 °C			
	FWHM	Tip Width	Ratio [%]	χ [%]	FWHM	Tip Width	Ratio [%]	χ [%]
CeO_2_	0.2362	0.2834	19.98	45.32	0.2187	0.2624	19.98	46.17
CGO10	0.2752	0.3302	19.98	42.67	0.2165	0.2598	20.00	43.90
CGO20	0.3033	0.3640	20.01	44.69	0.2381	0.2858	20.03	45.22
CGO30	0.3482	0.4179	20.02	40.19	0.3031	0.3637	19.99	40.64
CGO50					0.2949	0.3539	20.01	37.77

**Table 3:** Structural characteristics of (Ce)_1−x_(Gd)_x_(O)_2−x/2_ system samples, dried at 120 °C and calcined at 500 °C.

Sample	Space Group		Cell Parameter, Å				Crystallite Size, nm	
	120 °C	500 °C	120 °C		500 °C		120 °C	500 °C
			a [Å]	V [Å^3^]	a [Å]	V [Å^3^]		
CeO_2_	Fm-3m	Fm-3m	5.4166	158.92	5.4139	158.68	37	41
CGO10	Fm-3m	Fm-3m	5.4214	159.34	5.4219	159.39	31	34
CGO20	Fm-3m	Fm-3m	5.4291	160.02	5.4286	159.97	28	32
CGO30	Ia-3	Fm-3m	10.8619	1281.5	5.4308	160.17	24	26
CGO50	-	Ia-3	-		10.8707	1284.6	-	16

**Table 4:** Microstrain (ε) values of Ce_1−x_Gd_x_O_2−x/2_ samples before and after calcination as a function of Gd content. Arrows indicate the direction of change after calcination (↓ decrease, ↑ increase).

Sample	ε (120 °C)	ε (500 °C)	Change (%)
CeO_2_	9.41 × 10^−6^	1.40 × 10^−6^	−85% ↓
CGO10	7.37 × 10^−6^	2.98 × 10^−8^	−99.6% ↓
CGO20	2.57 × 10^−5^	1.44 × 10^−4^	+460% ↑
CGO30	5.28 × 10^−5^	3.68 × 10^−4^	+597% ↑
CGO50	—	1.83 × 10^−3^	(very high value)

**Table 5:** XPS results of quantitative composition on the surface.

Samples	O, at. %	Ce, at. %	Gd, at. %	Ce^3+^, %	Ce^4+^, %
CeO_2_	74.1	25.9	-	2.7	97.3
CG010	75.3	20.8	3.9	0.2	99.8
CG030	79.1	8.9	12.0	1.4	98.6
CG050	76.3	9.3	14.4	2.0	98.0

**Table 6:** Elemental composition of the dried and calcined samples from the (Ce)_1−x_(Gd)_x_(O)_2−x/2_ system investigated by EDS and EDXRF techniques.

Sample	EDXRF		EDS			
	Ce_500_, %	Gd_500_, %	Ce_120_, %	Gd_120_, %	Ce_500_, %	Gd_500_, %
CeO_2_	99.1	-	79.6	-	84.4	-
CGO10	87.6	11	72.7	9.0	73.9	9.2
CGO20	76.4	22.5	62.3	17.9	58.3	17.7
CGO30	64.9	33.2	56.3	26.5	56	22.6
CGO50	44.3	54	-	-	34.3	38.6

## Data Availability

The original contributions presented in this study are included in the article. Further inquiries can be directed to the corresponding author.
